# Clinical characteristics, treatment, and survival of thrombocytopenia induced by T-DM1 in early HER2-positive breast cancer

**DOI:** 10.3389/fonc.2025.1592440

**Published:** 2025-07-09

**Authors:** Fang Wang, Xiaosan Zhang, Xiuchun Chen, Lu Zhang, Zhenzhen Liu

**Affiliations:** Department of Breast Disease, Henan Breast Cancer Center, The Affiliated Cancer Hospital of Zhengzhou University and Henan Cancer Hospital, Zhengzhou, China

**Keywords:** HER2-positive breast cancer, T-DM1, thrombocytopenia, safety, effectiveness, survival

## Abstract

**Background:**

Trastuzumab emtansine (T-DM1) is widely used for treating both early and advanced HER2-positive breast cancer in China. Thrombocytopenia represents a major adverse event associated with T-DM1 during anti-tumor therapy. Therefore, further exploration is needed to predict and mitigate T-DM1-induced platelet count decrease.

**Materials and methods:**

We conducted a retrospective study utilizing electronic medical records from a single breast cancer center in Henan province. Clinicopathological characteristics and consecutive laboratory examination data were collected for all patients. A total of 63 patients treated with T-DM1 were categorized into two groups: Thrombocytopenia and Non-thrombocytopenia. Statistical analyses employed Chi-squared/Fishers exact test, F-test/Kruskal–Wallis test, logistic regression, and Kaplan–Meier methods.

**Results:**

A total of 63 HER2-positive patients receiving adjuvant T-DM1 were enrolled. A comparison between groups showed that patients in the Thrombocytopenia group were more frequently postmenopausal, had lymph node metastasis, and had undergone radiotherapy. Postmenopausal status was identified as a risk factor for T-DM1-induced thrombocytopenia. Grade ≥2 thrombocytopenia occurred in 30 patients (47.6%) and grade ≥3 thrombocytopenia occurred in 16 patients (25.4%). Patients treated with rhIL-1 1(recombinant human interleukin-11) or rhTPO (recombinant human thrombopoietin) required a longer time for platelet (PLT) recovery to ≥75 × 10^9^/L and ≥100 × 10^9^/L compared to those receiving TPO-Ras (thrombopoietin receptor agonists), although this difference was not statistically significant. The estimated 1-year invasive disease-free survival (IDFS) for all T-DM1-treated patients was 96.8%. The 1-year IDFS rates for the Thrombocytopenia and Non-thrombocytopenia groups were 100.0% and 93.9%, respectively, with no statistically significant difference observed.

**Conclusion:**

Our findings indicate that T-DM1-induced thrombocytopenia was manageable in this cohort of breast cancer patients. Postmenopausal status correlated with an increased risk of platelet count decrease. Different platelet-producing drugs demonstrated comparable effectiveness in managing thrombocytopenia.

## Introduction

Breast cancer is the most commonly diagnosed cancer among women globally and represents the leading malignancy in women in China (1). Human epithelial growth factor receptor 2 (HER2) is overexpressed in approximately 15%–30% of breast cancer cases and was historically associated with a poor prognosis (2). However, the survival outcomes of these patients have improved remarkably with the development of anti-HER2 therapies and are now comparable to those of patients with HER2-negative disease (3–6). Antibody drug conjugates (ADC) represent a class of targeted therapeutics comprising a cytotoxic agent conjugated to a monoclonal antibody via a stable covalent linker. Recent advances have established ADCs as a significant breakthrough in breast cancer treatment. They overcome resistance to trastuzumab and pertuzumab while reducing the systemic toxicity associated with high-dose chemotherapy (7).

Ado-trastuzumab emtansine (T-DM1) is an ADC consisting of the anti-HER2 antibody trastuzumab linked to the potent microtubule inhibitor DM1(emtansine) via a stable thioether linker (8). Based on the pivotal EMILIA and TH3RESA trials (3, 9), T-DM1 became the standard second-line therapy for HER2-positive advanced breast cancer previously treated with trastuzumab and taxane. Due to its significant efficacy, T-DM1 was subsequently evaluated in early-stage breast cancer. In May 2019, the FDA (Food and Drug Administration) approved T-DM1 for adjuvant treatment of HER2-positive early breast cancer based on the results of the KATHERINE trial (10). T-DM1 received approval from the NMPA (National Medical Products Administration) in China in 2020 and was included in the national medical insurance reimbursement in 2023 leading to its widespread clinical use since then.

Thrombocytopenia is one of most common adverse events induced by T-DM1 treatment and represents the primary reason for dose reduction and treatment discontinuation (3, 11–13). The overall incidence of thrombocytopenia is approximately 39% in Asian population, with grade 3 or higher events occurring in approximately 20% of the patients (14). Therefore, this study aimed to explore the clinical characteristics and treatment of thrombocytopenia induced by T-DM1 in the real world, as well as to evaluate the safety and efficacy of T-DM1 treatment in patients with early breast cancer.

## Materials and methods

### Study population

**Figure 1:**
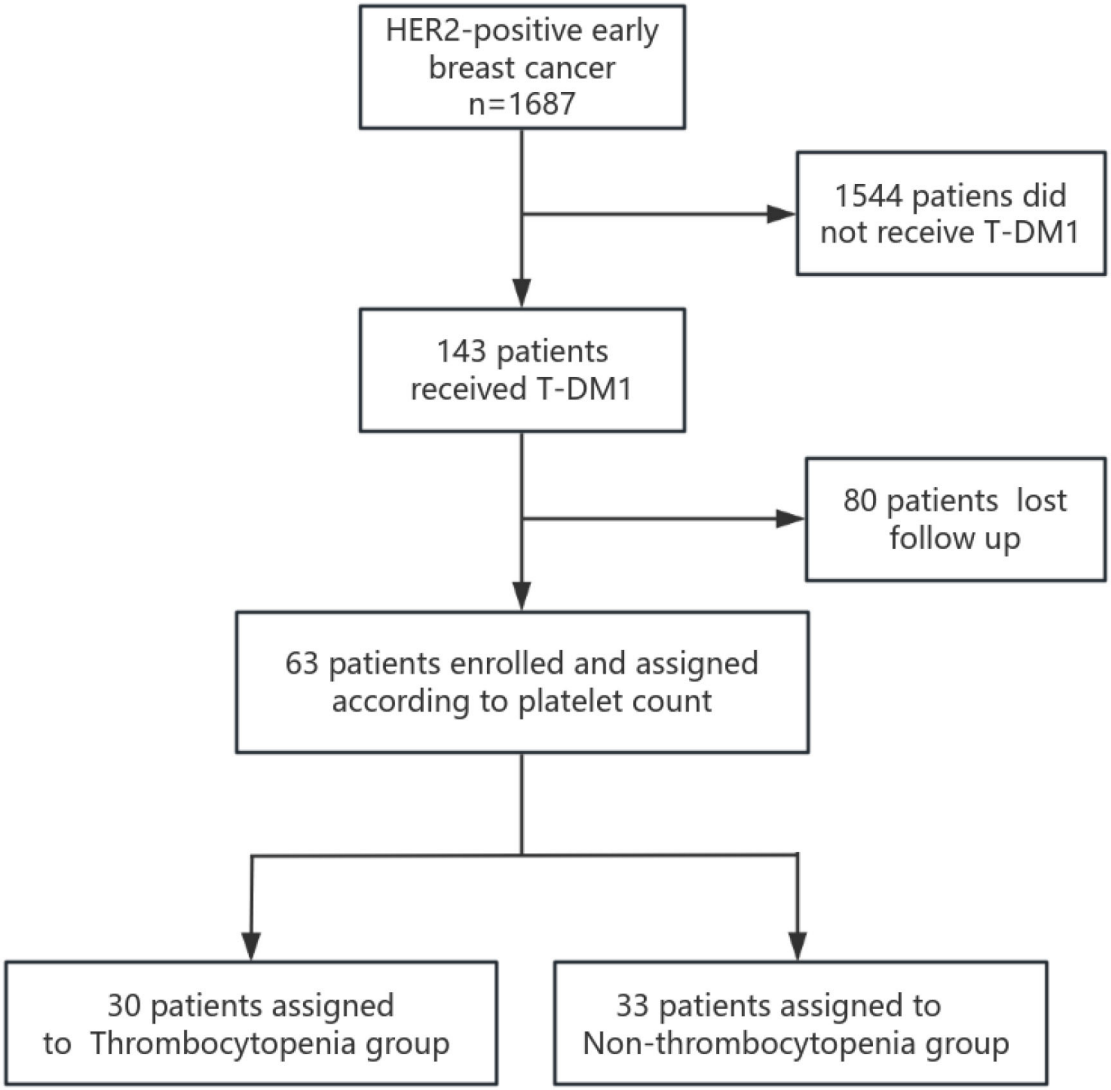
Study flowchart.

We conducted a retrospective analysis of 143 patients with primary HER2-positive early breast cancer who were diagnosed and treated with T-DM1 at the Affiliated Cancer Hospital of Zhengzhou University and Henan Cancer Hospital between January 2022 and December 2023. Electronic medical records and pathology reports of all patients were reviewed. Data extracted included patient age, sex, menstrual status, tumor size, axillary lymph node status, clinical stage, pathological stage, histologic grade, HER2 expression status, estrogen receptor (ER) and progesterone receptor (PR) expression levels, Ki-67 staining, pathological results of biopsy, resected surgical specimens and treatment methods (endocrine therapy, radiotherapy, and chemotherapy), and survival information. Patients with clinical stage IV disease, a history of previous malignancy, or unavailable clinicobiological data were excluded. Additionally, patients with a history of hematological disease or incomplete laboratory data during T-DM1 treatment were excluded. Finally, 63 patients met the inclusion criteria and were enrolled in this study (**Figure 1**).

### Definitions and outcomes

HER2 positivity was defined as an immunohistochemistry (IHC) score of 3+ or IHC 2+ with positive fluorescence *in situ* hybridization (FISH). All patients received T-DM1 administered intravenously at a dose of 3.0 or 3.6 mg/kg every 21 days for up to 14 cycles. Adverse events (AEs) were documented following T-DM1 and graded according to the Common Terminology Criteria for Adverse Events (CTCAE) Version 5.0. Thrombocytopenia referred to a platelet count below 100 × 10^9^/L in the peripheral blood. Grades for thrombocytopenia were assigned as follows: grade 1: 75 to <100 × 10^9^/L, grade 2: 50 to <75 × 10^9^/L, grade 3: 25 to <50 × 10^9^/L, and grade 4: <25 × 10^9^/L (15). As grade <2 thrombocytopenia typically did not necessitate affecting the timely and sufficient administration of T-DM1, patients were divided into two groups based on platelet nadir: a Thrombocytopenia group (≥Grade 2 thrombocytopenia) and a Non-thrombocytopenia group (platelet counts consistently within normal range or Grade 1 thrombocytopenia only). First thrombocytopenia was defined as the first time detecting the platelet count below 75 × 10^9^/L during T-DM1 treatment. Invasive disease-free survival (IDFS) was defined as the time from randomization until the first occurrence of any of the following events: ipsilateral invasive breast tumor recurrence, ipsilateral locoregional invasive breast cancer recurrence, contralateral invasive breast cancer, distant recurrence, or death from any cause. All patients were followed up from June 2022 to December 2024, with a median follow-up duration of 17 months.

### Statistics

Statistical analyses were performed using SPSS software (version 26.0). Categorical variables are presented as frequencies and percentages. Continuous variables are reported as mean ± standard deviation (SD) or median with interquartile range (IQR) based on their distribution. Differences in categorical variables between groups were assessed using the Chi-square or Fisher’s exact tests, as appropriate. Normally distributed continuous variables were compared using analysis of variance (ANOVA; reported as F-test results), while non-normal distributed continuous variables were compared using the Kruskal–Wallis test. Logistic regression was employed to estimate the association between potential risk factors and the occurrence of thrombocytopenia (≥Grade 2). Survival outcomes was analyzed using the Kaplan–Meier method.

## Results

A total of 143 patients, diagnosed with early breast cancer and who received T-DM1, were screened, and 63 patients were included in the final analysis comprising 30 patients in the Thrombocytopenia group and 33 patients in the Non-thrombocytopenia group. Baseline demographics and clinicopathological characteristics by platelet count status are summarized in **Table 1**. A comparison between groups showed that patients in the Thrombocytopenia group were more postmenopausal, and the difference was statistically significant (46.7% vs. 21.2%, p = 0.032). Lymph node metastasis was significantly more prevalent in the Thrombocytopenia group compared to the Non-thrombocytopenia group (73.3% vs. 48.5%, respectively, p = 0.044). Patients in the Thrombocytopenia group had undergone radiotherapy significantly more often compared with the Non-thrombocytopenia group (100.0% vs. 81.8%, p = 0.043). There was no significant difference between the two groups in terms of age, ECOG status, BMI, tumor size, histologic grade, ki-67 expression, ER expression, PR expression, HER2 receptor expression, type of breast surgery, neoadjuvant chemotherapy regimen, clinical stage, pathological stage, Miller–Payne grade, hormonal therapy, radiation therapy, radiation time, platelet count at baseline, white blood cells at baseline, T-DM1 starting dose, complicated with thrombosis, previous ≥III-grade myelosuppression. In the Thrombocytopenia group, the number of patients with baseline platelet count of 100–219 × 10^9^/L, 220–300 × 10^9^/L, and >300 × 10^9^/L were 17 (56.7%), 9 (30.0%), and 4 (13.3%), respectively. In the Non-thrombocytopenia group, the number of patients with baseline platelet count of 100–219 × 10^9^/L, 220–300 × 10^9^/L, >300 × 10^9^/L were 14 (42.4%), 11 (33.3%), and 8 (24.2%),respectively. In the Thrombocytopenia group, the T-DM1 starting dose at 3.0 and 3.6 mg/kg accounted for 13.3% and 86.7%, respectively. In the Non-thrombocytopenia group, the T-DM1 starting dose at 3.0 and 3.6 mg/kg accounted for 27.3% and 72.7%, respectively. The modification of the T-DM1 starting dose mainly was based on previous myelosuppression in the neoadjuvant therapy and current patients’ physical condition. The logistic regression analysis suggested that postmenopausal (OR =3.158; p = 0.047) was the risk factor for thrombocytopenia in breast cancer patients receiving T-DM1 (**Table 2**).

**Table 1:** Patients clinicopathologic characteristics at baseline.

Characteristic	Thrombocytopenia (n=30)	Non-thrombocytopenia (n=33)	p-value
Age (year) Median (range) <50 ≥50 ECOG performance status <1 ≥1 BMI ≤24kg/m^2^ >24kg/m^2^ Menopausal status Premenopausal Postmenopausal Tumor size (cm) ≤2 >2 Lymph node invasion Positive Negative Clinical stage before neoadjuvant therapy I IIA IIB IIIA IIIB IIIC Pathological stage after surgery I IIA IIB IIIA IIIB IIIC Histologic grade I II III Unknown Ki-67 expression <30% ≥30% Estrogen receptor expression <1% 1%-10% >10% Progesterone receptor expression <1% ≥1% HER2 receptor expression 2+ 3+ Surgery method Mastectomy+ALND Mastectomy+SLNB Breast-conserving surgery+ALND Breast-conserving surgery+SLNB NACT regimen T/PCb±HP T/P+HP AC-THP TEC Miller-Payne grade G1-3 G4-5 Hormonal therapy Yes No Radiation therapy Yes No Radiation time Before T-DM1 Combined with T-DM1 Platelet count at baseline (×10^9^/L) 100-219 220-300 >300 White Blood Cells at baseline <LLN ≥LLN T-DM1 starting dose 3.0kg/mg 3.6kg/mg Complicated with thrombosis Yes No Previous ≥III grade myelosuppression Yes No	50 (33-64) 15 (50.0%) 15 (50.0%) 19 (63.3%) 11 (36.7%) 13 (43.3%) 17 (56.7%) 16 (53.3%) 14 (46.7%) 20 (66.7%) 10 (33.3%) 22 (73.3%) 8 (26.7%) 2 (6.7%) 5 (16.7%) 15 (50.5) 4 (13.3%) 3 (10.0%) 1 (3.3%) 7 (23.3%) 11 (36.7%) 2 (6.7%) 4 (13.3%) 0 (0.0%) 6 (20.0%) 1 (3.3%) 13 (43.3%) 3 (10.0%) 13 (43.3%) 4 (13.3%) 26 (86.7%) 5 (16.7%) 3 (10.0%) 22 (73.3%) 10 (33.3%) 20 (66.7%) 16 (53.3%) 14 (46.7%) 24 (80.0%) 0 (0.0%) 5 (16.7%) 1 (3.3%) 21 (70.7%) 7 (23.3%) 1 (3.3%) 1 (3.3%) 19 (63.3%) 11 (36.7%) 24 (80.0%) 6 (20.0%) 30 (100.0%) 0 (0.0%) 4 (13.3%) 26 (86.7%) 17 (56.7%) 9 (30.0%) 4 (13.3%) 4 (13.3%) 26 (86.7%) 4 (13.3%) 26 (86.7%) 3 (10.0%) 27 (90.0%) 3 (10.0%) 27 (90.0%)	44 (28-65) 21 (63.6%) 12 (36.4%) 27 (81.8%) 6 (18.2%) 13 (39.4%) 20 (60.6%) 26 (78.8%) 7 (21.2%) 25 (75.8%) 8 (24.2%) 16 (48.5%) 17 (51.5%) 4 (12.1%) 12 (36.4%) 12 (36.4%) 5 (15.2%) 0 (0.0%) 0 (0.0%) 16 (48.5%) 6 (18.2%) 4 (12.1%) 5 (15.2%) 0 (0.0%) 2 (6.1%) 0 (0.0%) 9 (27.3%) 3 (9.1%) 21 (63.6%) 4 (12.1%) 26 (87.9%) 10 (30.3%) 4 (12.1%) 19 (57.6%) 14 (42.4%) 19 (57.6%) 16 (48.5%) 17 (51.5%) 21 (63.6%) 5 (15.2%) 6 (18.2%) 1 (3.0%) 26 (78.8%) 6 (18.2%) 1 (3.0%) 0 (0.0%) 20 (60.6%) 13 (39.4%) 21 (63.6%) 12 (36.4%) 27 (81.8%) 6 (18.2%) 3 (11.1%) 24 (88.9%) 14 (42.4%) 11 (33.3%) 8 (24.2%) 5 (15.2%) 28 (84.8%) 9 (27.3%) 27 (72.7%) 3 (9.1%) 30 (90.9%) 6 (18.2%) 27 (81.8%)	0.275 0.099 0.751 **0.032** 0.425 **0.044** 0.155 0.112 0.313 1.000 0.418 0.458 0.701 0.119 0.817 0.824 0.151 **0.043** 1.000 0.431 1.000 0.172 1.000 0.571

ECOG, Eastern Cooperative Oncology Group; BMI, body mass index; HER2, human epidermal growth factor receptor 2; SLNB, sentinel lymph node biopsy; ALND, axillary lymph node dissection; NACT, neoadjuvant chemotherapy; T, docetaxel; P, paclitaxel; Cb, carboplatin; H, trastuzumab; P, pertuzumab; A, anthracycline; C, cyclophosphamide; E, epirubicin; LLN, Lower limit of normal.

Bold values represent statistically significant difference (p<0.05).

**Table 2:** The relative risk factors for thrombocytopenia.

Variable	*χ ^2^*	OR	p-value	95% CI
Postmenopausal	3.951	3.158	**0.047**	1.016–9.812
Lymph node invasion	3.48	2.837	0.062	0.949–8.483

OR, odds ratio; CI, confidential interval.

Bold values represent statistically significant difference (p<0.05).

**Table 3** outlines the clinical characteristics of thrombocytopenia induced by T-DM1. Thrombocytopenia was first reported following a median of 2 (1–9) cycles of T-DM1. The median time of the first thrombocytopenia days after receiving T-DM1 was 7 (4–20) days. The median time of the first thrombocytopenia duration was 7 (3–14) days. Grade ≥2 thrombocytopenia occurred in 30 patients (47.6%) and grade ≥3 thrombocytopenia occurred in 16 (25.4%) patients. Hemorrhage events occurred in 7 (11.1%) patients. Five (7.9%) patients required dose reductions to 3.0 or 2.4 mg/kg. Treatment delay was required in 12 (19.0%) patients. T-DM1 was discontinued due to thrombocytopenia in 5 (7.9%) patients. The number of patients complicated with leukopenia, neutropenia, anemia, hepatotoxicity, hypokalemia, and splenomegaly were 18 (28.6%), 10 (15.9%), 5 (7.9%), 21 (33.3%), 15 (23.8%), and 4 (6.3%), respectively.

**Table 3:** The clinical characteristics of thrombocytopenia induced by T-DM1.

Characteristic	Median (minimum–maximum)	N (n = 63)	%
Cycle of first occurrence of thrombocytopenia	2 (1–9)		
First thrombocytopenia days after T-DM1	7 (4–20)		
First thrombocytopenia duration	7 (3–14)		
≥2 grade thrombocytopenia		30	47.6
≥3 grade thrombocytopenia		16	25.4
Hemorrhage		7	11.1
AEs leading to dose reduction		5	7.9
AEs leading to treatment delay		12	19
AEs leading to treatment discontinuation		5	7.9
Complicated with leukopenia		18	28.6
Complicated with neutropenia		10	15.9
Complicated with anemia		5	7.9
Complicated with hepatotoxicity		21	33.3
Complicated with hypokalemia		15	23.8
Complicated with splenomegaly		4	6.3

AEs, adverse events.

**Table 4** describes the efficacy of different platelet-producing drugs including rhIL-11 (recombinant human interleukin-11), rhTPO (recombinant human thrombopoietin), and TPO-Ras (thrombopoietin receptor agonists) in the first thrombocytopenia. A comparison between groups revealed that patients in the rhIL-11 and rhTPO group had undergone a longer time of PLT recovery to ≥75 × 10^9^/L and PLT recovery to ≥100 × 10^9/^L; however, the difference was not statistically significant. The patients receiving TPO-RAs had more ≤7 days of PLT recovery to ≥75 × 10^9^/L than the patients receiving rhIL-11 and rhTPO, but the difference was not statistically significant. Two (3.17%) patients received platelet transfusions. Other combined platelet-producing drugs included caffeic acid tablets, platelet booster capsule, levomisole, danazol, and calcitriol, which were similar between the groups.

**Table 4:** The efficacy of different platelet-producing drugs in the first thrombocytopenia.

Efficacy	rhIL-11 (n = 7)	rhTPO (n = 18)	TPO-RAs (n = 5)	p-value
The time of PLT recovery to ≥75 × 10^9^/L	7 (3–12)	7 (3–14)	4 (3–14)	0.532
The time of PLT recovery to ≥100 × 10^9^/L	7 (5–12)	7 (5–14)	4 (3–14)	0.24
≤7 days of PLT recovery to ≥75 × 10^9^/L				1
Yes	5 (71.4%)	14 (77.8%)	4 (80.0%)	
No	2 (28.6%)	4 (22.2%)	1 (20.0%)	
Combined with other treatments				0.365
Yes	3 (42.8%)	5 (27.8%)	0 (0.0%)	
No	4 (57.1%)	13 (72.2%)	5 (100.0%)	

rhIL-11, recombinant human interleukin-11; rhTPO, recombinant human thrombopoietin; TPO-RAs, thrombopoietin receptor agonists; PLT, platelet.

**Figure 2:**
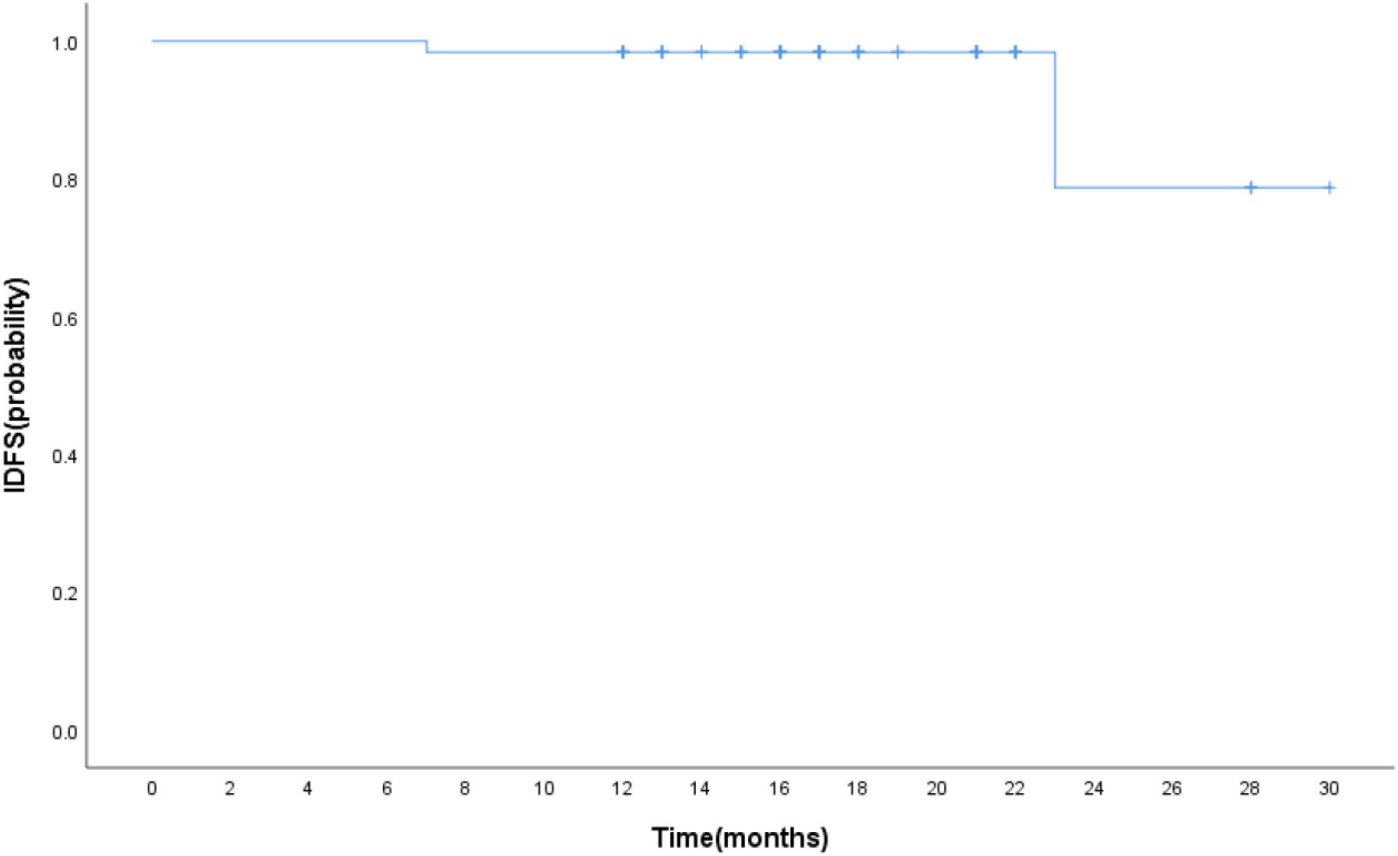
Kaplan–Meier estimates of invasive disease-free survival in patients who received T-DM1.

**Figure 3:**
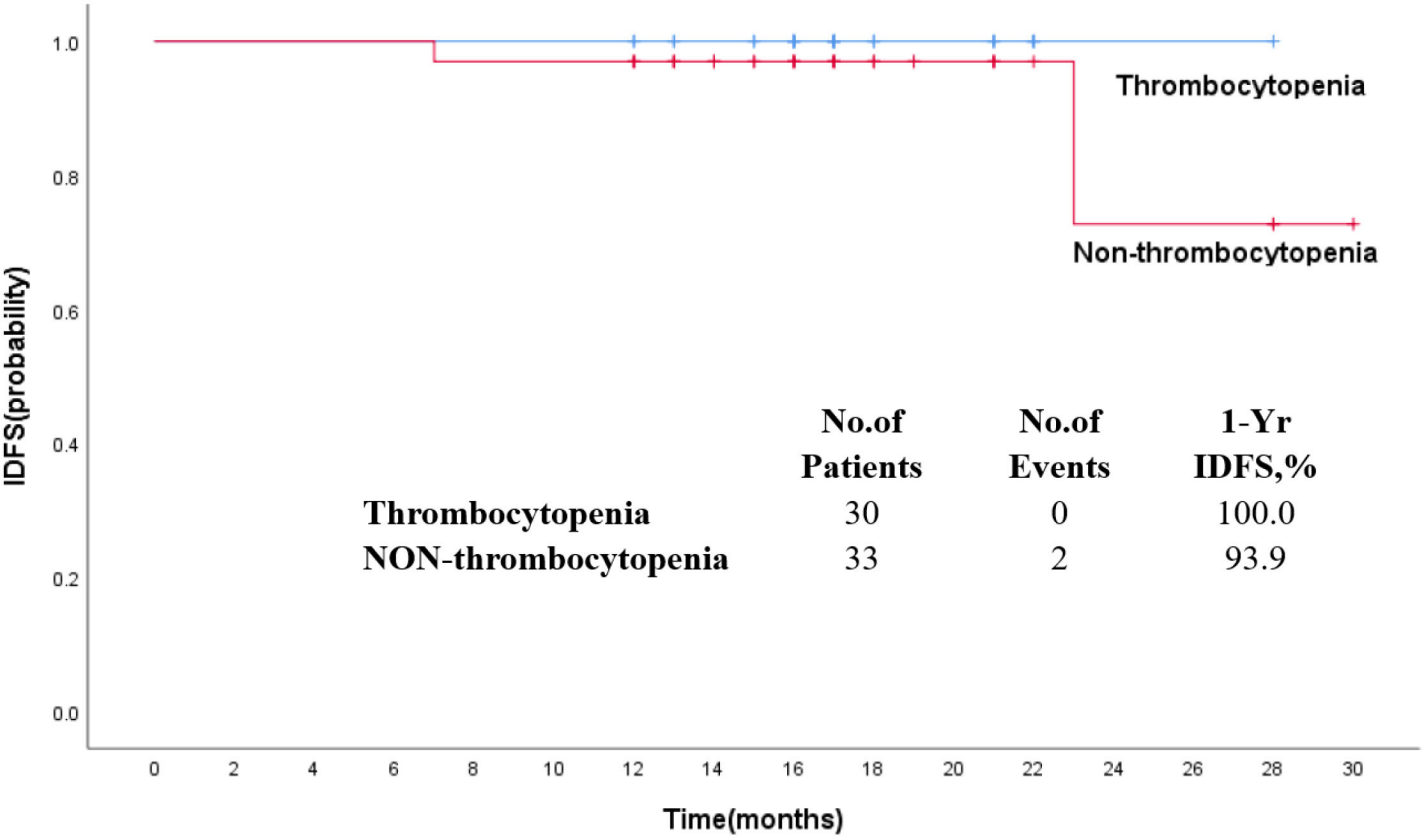
Kaplan–Meier estimates of invasive disease-free survival in patients who received T-DM1 with thrombocytopenia or non-thrombocytopenia.

The median follow-up was 17 months. The estimated 1-year IDFS was 96.8% for all patients treated with T-DM1 (**Figure 2**). The 1-year IDFS of Thrombocytopenia and Non-thrombocytopenia was 100.0% and 93.9, respectively. The difference between both groups did not meet the threshold for statistical significance (p = 0.291) (**Figure 3**). Overall, there were two events of recurrence in the Non-thrombocytopenia group, of which one patient was dead.

## Discussion

As we all know, T-DM1 demonstrated a more favorable safety profile, with thrombocytopenia and hepatotoxicity being the main adverse events (AEs).In the KATHERINE trial, T-DM1 was used for residual early HER2-positive BC, and 5.7% of the patients developed Grade 3 or higher thrombocytopenia. In the EMILIA trial, thrombocytopenia was the most common AE in the patients treated with T-DM1, and the incidence of this AE (Grade 3 and above) was higher (12.9%) than that of the capecitabine plus lapatinib group (16). A meta-analysis of 29 included studies demonstrated that the total incidence of thrombocytopenia induced by T-DM1 was 20%–32.2%,and the 3/4 Grade was 5%–11.9%, where the incidence of Asian patients was 20.0% (14). Another study also found that Asian patients treated with T-DM1 have a higher incidence of thrombocytopenia (Grade 3 and above) than non-Asian patients (44.4% vs. 10.6%) (17). A real-world study in China investigated that the ≥3 grade thrombocytopenia accounted for 4.3% of metastatic BC and 29.0% of early BC groups, respectively (18). So far, our observation is the first retrospective study of patients with decreased platelet count induced by T-DM1 in China. In our analysis of 63 HER2-positive early breast cancer patients treated with T-DM1, we found that grade ≥2 thrombocytopenia occurred in 30 (47.6%) patients, and grade ≥3 thrombocytopenia occurred in 16 (25.4%) patients, largely consistent with the results mentioned above.

Many studies tried to identify relative risk factors for the development of thrombocytopenia in patients treated with T-DM1. Zhang et al. reported that a meta-analysis of 29 studies on T-DM1 demonstrated that Asian patients have a higher risk of developing thrombocytopenia after receiving T-DM1 than non-Asian patients. They also found that the dose of TDM1 was associated with the development of thrombocytopenia (14). Mamounas et al. found that the incidence of all-grade and grade 3 thrombocytopenia was higher in previous platinum-treated patients than in patients without platinum-treated therapy in a phase III clinical trial (19). Natansh et al. presented a predictive model for grade ≥3 thrombocytopenia optimally defined by race and pre-treatment platelet count. Asians and a platelet count of 100–220 × 10^9^/L had a higher incidence of thrombocytopenia (20). In the subgroup analyses from KATHERINE (19), a platinum-containing neoadjuvant regimen had been administered to 19.9% of patients who received T-DM1 and was associated with a higher incidence of grade 3 thrombocytopenia than in those without platinum. We first identified that postmenopausal, lymph node invasion, and receiving radiation therapy were potential associations with thrombocytopenia reduced by T-DM1, but there was no significant difference in the previous platinum-treated therapy and pre-treatment platelet count between the two groups. The inconsistent results may stem from the different race, disease stage, platinum-containing regimen, T-DM1 dosing, or other unmeasured factors. Notably, logistic regression analysis identified postmenopausal status as an independent risk factor for thrombocytopenia, a finding not previously reported. This association may be potentially attributable to the older age and weaker bone marrow function of postmenopausal patients. Furthermore, we observed that lymph node metastasis was significantly more prevalent in the Thrombocytopenia group. This result could be associated with the more sufficient T-DM1 starting dose and radiotherapy after surgery of patients with lymph node metastasis.

Recently, there were some reports with regard to early and advanced breast cancer patients treated with T-DM1. Two (6.5%) in the early breast cancer group experienced a dose reduction due to a platelet count decrease (18). For the majority of patients, grade 3 or above thrombocytopenia developed during the first two cycles of T-DM1 treatment, and 10 (2.0%) patients were not able to continue T-DM1 treatment because of thrombocytopenia. The any grade and 3 or 4 grade incidence of bleeding events with T-DM1 was 29.8% and 1.4%, respectively (16). In The Royal Marsden Hospital, T-DM1 was discontinued, and the dose was reduced due to thrombocytopenia in 4 (3.1%) and 8 (6.3%) patients, respectively (21). T-DM1-associated thrombocytopenia was most commonly grade 1 or 2 in severity, with the platelet count decrease happening by day 8 (17). The decreased platelet count (4.2%) was the most common adverse event leading to drug discontinuation and 1 (0.14%) patient died from an intracranial hemorrhage after decreased platelet count in the T-DM1 group (22). There were 43 (15%) patients who experienced a dose interruption, 67 (22%) patients who experienced a dose reduction, and 47 (15%) patients who discontinued T-DM1. Median time to grade 3 or 4 thrombocytopenia was 12 days, and 70% of grade 3 or 4 events developed within the first two cycles of T-DM1 (20). Another retrospective study detected that the median therapy cycle in which thrombocytopenia first appeared was in cycle 3, and 66.7% of the patients developing thrombocytopenia were in the first four cycles. Except for thrombocytopenia, the common adverse events include hepatotoxicity (42.87 and 37.7% for AST and ALT, respectively), neutropenia (14.5%), hypokalemia (9.4%), and anemia (38%) (23). Our data indicated that the median number of cycles to the first thrombocytopenia was 2 (1–9), similar to the previous report. However, the median number of days to the first thrombocytopenia after T-DM1 treatment was 7 (4–20), lower than the 12 days observed by Natansh et al. The any grade incidence of bleeding events following thrombocytopenia was 11.1%, and there were no patients who experienced – grade 3–4 hemorrhage. The dose of T-DM1 was reduced, delayed, and withdrawn because of platelet count decrease in 5 (7.9%), 12 (19.0%), and 5 (7.9%) patients. Other complications with thrombocytopenia included leukopenia (28.6%), neutropenia (15.9%), anemia (7.9%), hepatotoxicity (33.3%), hypokalemia (23.8%), and splenomegaly (6.3%), which are partly consistent with the data reported before.

At present, only rhTPO and rhIL-11 have been approved by the NMPA to treat cancer treatment-induced thrombocytopenia (CTIT) in mainland China, but many studies have confirmed the efficiency and safety of TPO-RA drugs in non-hematologic malignancies. There is no relevant study on the use of platelet-producing drugs in patients with thrombocytopenia secondary to T-DM1. A real-world study on thrombopoietic agents for patients with CTIT in China showed that most patients were treated with either rhTPO alone (49.3%) or rhIL-11 alone (27.0%), and the most common combination therapy applied was rhIL-11 and rhTPO (10.9%) (24). They suggested that rhTPO was related to a lower proportion of platelet recovery and platelet transfusion than that of rhIL-11. However, no significant difference was observed in the time taken to achieve a platelet count of >100 × 10^9^/L among the three platelet-producing agents, similar to the results observed by us. In another treatment of CTIT, rhTPO and rhIL-11 also showed a similar effectiveness including the platelet compliance rate, mean days of medication, and median days of compliance (25).

In the KATHERINE clinical trial, the percentages of 3 years of invasive disease-free survival (IDFS) were higher in the T-DM1 group (88.3%) than in the trastuzumab group (77.0%), and there was a significant difference between the two groups (p < 0.001) (22). A subgroup analyses from KATHERINE proved that the IDFS benefit of receiving adjuvant T-DM1 compared with trastuzumab was similar regardless of prior neoadjuvant AC use (19). A neoadjuvant treatment with T-DM1 demonstrated that 3-year IDFS event-free rates were 93.0% with T-DM1 + P and 92.0% with TCH + P, with no significant difference (26). T-DM1, as adjuvant treatment for patients with stage I HER2-positive breast cancer, resulted in 11 IDFS events in the T-DM1 group, consistent with a 5-year IDFS of 97.0% (27). The association of thrombocytopenia due to T-DM1 with survival is currently unknown in early-stage breast cancer. In our present study, the estimated 1-year IDFS was 96.8% for all patients treated with T-DM1, and no statistical significant difference between the Thrombocytopenia and Non-thrombocytopenia arms was observed.

Our research has some limitations. First, it is a retrospective and single-center study, which may cause heterogeneity, differences in patient timing, and statistical bias in the analysis. Additionally, because of the relatively short follow-up time, longer IDFS and OS are missing. In the future, more findings would need to be confirmed in larger, multicenter prospective cohorts.

## Conclusion

The findings indicated that thrombocytopenia induced by T-DM1 was safe for early breast cancer patients. Postmenopausal status was correlated with the occurrence of platelet count decrease during T-DM1 treatment. The different platelet-producing drugs showed similar effectiveness for thrombocytopenia.

## Data Availability

The original contributions presented in the study are included in the article/supplementary material. Further inquiries can be directed to the corresponding author.
